# Total Iron Measurement in Human Serum With a Novel Smartphone-Based Assay

**DOI:** 10.1109/JTEHM.2020.3005308

**Published:** 2020-06-26

**Authors:** Michael Serhan, David Jackemeyer, Mindy Long, Mark Sprowls, Ismael Diez Perez, Wolfgang Maret, Fang Chen, Nongjian Tao, Erica Forzani

**Affiliations:** 1 Arizona State University 7864 TempeAZ85287USA; 2 King’s College London 4616 LondonWC2R 2LSU.K.

**Keywords:** Biosensors, chemical and biological sensors, sensor systems, image color analysis, image capture and iron

## Abstract

*Background*: Abnormally low or high blood iron levels are common health conditions worldwide and can seriously affect an individual’s overall well-being. A low-cost point-of-care technology that measures blood iron markers with a goal of both preventing and treating iron-related disorders represents a significant advancement in medical care delivery systems. *Methods:* A novel assay equipped with an accurate, storable, and robust dry sensor strip, as well as a smartphone mount and (iPhone) app is used to measure total iron in human serum. The sensor strip has a vertical flow design and is based on an optimized chemical reaction. The reaction strips iron ions from blood-transport proteins, reduces Fe(III) to Fe(II), and chelates Fe(II) with ferene, with the change indicated by a blue color on the strip. The smartphone mount is robust and controls the light source of the color reading App, which is calibrated to obtain output iron concentration results. The real serum samples are then used to assess iron concentrations from the new assay, and validated through intra-laboratory and inter-laboratory experiments. The intra-laboratory validation uses an optimized iron detection assay with multi-well plate spectrophotometry. The inter-laboratory validation method is performed in a commercial testing facility (LabCorp). *Results*: The novel assay with the dry sensor strip and smartphone mount, and App is seen to be sensitive to iron detection with a dynamic range of 50 – $300~\mu \text{g}$/dL, sensitivity of 0.00049 a.u/$\mu \text{g}$/dL, coefficient of variation (CV) of 10.5%, and an estimated detection limit of $\sim 15~\mu \text{g}$/dL These analytical specifications are useful for predicting iron deficiency and overloads. The optimized reference method has a sensitivity of 0.00093 a.u/$\mu \text{g}$/dL and CV of 2.2%. The correlation of serum iron concentrations (N = 20) between the optimized reference method and the novel assay renders a slope of 0.95, and a regression coefficient of 0.98, suggesting that the new assay is accurate. Last, a spectrophotometric study of the iron detection reaction kinetics is seen to reveal the reaction order for iron and chelating agent. *Conclusion:* The new assay is able to provide accurate results in intra- and inter- laboraty validations, and has promising features of both mobility and low-cost manufacturing suitable for global healthcare settings.

## Graphical abstract

We present a colorimetric assay determining the concentration of iron in a 30 μL serum sample in five minutes, all from the convenience of the smartphone. The 3D-printed system includes the sensor cartridge (sandwiching the sensing membranes), detection chamber to fix the position of the sensor strip, and iPhone camera mount for reproducible imaging conditions. The color formed on the sensing surface correlates with the concentration of iron in the sample and is determined via RGB analysis with an iOS app.

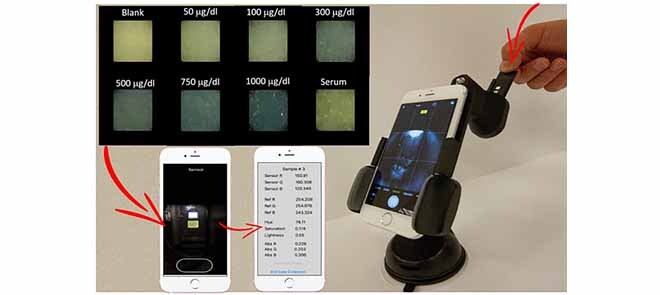

## I. Introduction

Iron is essential in maintaining health in humans due to reliance on oxygen binding (heme), electron transport (energy production), and as a catalyst of hundreds of enzymes (redox and immune control) [1], [2]. Iron metabolism is guided by a complex set of genetically regulated processes for storage, transportation, and dietary uptake during feeding, thereby providing sufficient iron to all cells regardless of fluctuating dietary quantities, blood losses, or gains via transfusions [1]–[3]. However, both iron deficiency and iron overload can result from a variety of dysfunctions, potentially leading to permanent damage of organ systems such as the liver and brain [4], [5]. Iron *deficiency* is a frequent concern for those with blood loss, including healthy menstruating females, and in all populations with limited access to proper nutrition via whole food diets or even with highly processed/refined foods with appropriate fortification [4], [6]–[8]. On the other hand, iron *overload* is a threat, primarily to those (genetically) inheriting the so-called hemochromatosis genes from both parents, the recessive “High Iron” (HFE) C282Y allele with incidence of 1 in ~300 people of northern European decent. This disease is difficult to screen due to vague symptoms (e.g., fatigue), but its progress leads to parenchymal damage in various organs and liver disease, pancreatic impairment (diabetes), heart arrhythmias or failure, and neurodegenerative disorders of the brain [5], [9]–[11]. In fact, over several years, the amount of stored iron in blood can reach 10+ grams (from a normal of 3–4 grams), saturating the iron transporters, and over-filling or “spilling-over” storage into an increasingly toxic labile intracellular pool [3], [5], [11]. Thus, iron distribution within the overloaded must be tightly regulated by medication or iron-removal strategies to avoid loss of function from irreversible damage to the organs.

A well-cared for patient’s annual physical exam should include determination of iron metabolism biomarkers, but unfortunately, due to cost, only two proxies for iron metabolism, hemoglobin and red blood cells, are commonly assessed, both poor markers for iron overload thereby leaving hemochromatosis as a disorder typically detected late in life when irreversible damage on organs is detected. Furthermore, prevention and interventions (e.g., supplementation) to address iron imbalances are costly, thereby leaving individuals at risk of prolonged state of deficiency or overload [3], [10], [11]. Total iron, i.e., total bound iron binding capacity (TIBC) or unbound (UIBC), and ferritin (iron storage protein) are clinically validated blood-derived biomarkers of iron deficiency or overload [3], [12], [13]. In this publication, we focus on measuring total iron because it is i) the most direct metabolite of the panel and ii) is measured when determining the UIBC or TIBC, which calculate percent saturation of the transferrin (transporters of iron). Our goal is to create a tool for globally screening of iron deficiencies or overloads. Measurement of all the clinically valid biomarkers is time-consuming, expensive, and painful requiring venous blood draw, temperature-controlled storage and shipping, and use of laboratory-based expertise and instruments such as spectrophotometry [14], [15].

Due to the above-mentioned limitations, we present here a low-cost novel assay for detection of iron based on storable, dry, and disposable sensor strips and a smartphone mount and application to reduce the need of laboratory space and special instrumentation and to conduct all analyses at room temperature at a low manufacturing and end-user cost. We choose the smartphone as a detection platform since the ever-increasing number of smartphone owners (3 billion+ as of 2020) opens the possibility to deliver low-cost detectors everywhere with proven capabilities of complex imaging algorithms for clinical applications [16]–[23].

## II. Experimental Methods

### A. Reference Method for Iron Detection

A reliable certified laboratory reference method for iron quantification is a spectrophotometric assay that includes a “reagent A”, containing 200 mM citric acid, 34 mM ascorbic acid, 100 mM thiourea, and surfactant; “reagent B”, containing ferene at >3 mM, and the tested sample with final volume ratios of 5:1:1 [24]. The lab protocol begins with whole blood samples processed to isolate serum or heparinized plasma, then processed with “reagent A” to strip Fe (III) from transferrin with citric acid, followed by reducing Fe(III) to Fe(II) with ascorbic acid, and finally the addition of “reagent B” to chelate Fe(II) to the chromophore ferene [24]. The recommended incubation time for the final reaction is thirty minutes [24]. Ferene is chosen because of the direct proportionality of iron concentrations to absorbance values from the ferrous complex and high absorptivity at 575-600 nm range [14], [25], [26]. Thiourea is included to quench Cu(II), a known interferent in blood iron detection [27]. A surfactant is used in order to contribute to reaction homogeneity.

### B. Optimized Method for Iron Detection

Two issues with the current reference method encouraged us to optimize it: 1) a need to avoid apparent protein precipitation during the incubation of serum samples, and 2) a need to increase sensitivity to assure accuracy of detection for low iron concentrations. To address these issues, we: 1) removed the surfactant causing sample turbidity and unusual high absorbance values and 2) reduced the volume of “reagent A” to a ratio of 3:1:1 to increase relatively higher iron and ferene concentrations (see section III, Results and Discussion) The changes resulted in the creation of what we refer to as an “optimized reference method”. **Table 1** shows the resulting final molar ratios of the original reference method versus the optimized reference method for use in the spectrophotometer. Detailed rationale for sensor strip chemistry ratios is given in section *F. Sensor strip design* in experimental methods.

**TABLE 1:** Comparison of Molar Concentration Ratios of Reagents to Iron for the Three Analytical Methods Used

Reagent	Orig. Ref. Method	Opt. Ref. Method	Sensor Method
Ascorbic acid	$9.5\times 10^{3}$	$5.7\times 10^{3}$	$2.7\times 10^{3}$
Citric acid	$55\times 10^{3}$	$34\times 10^{3}$	$16\times 10^{3}$
Thiourea	$28\times 10^{3}$	$17\times 10^{3}$	$8.0\times 10^{3}$
Ferene	$2.2\times 10^{2}$	$2.2\times 10^{2}$	$3.2\times 10^{2}$
Iron	1.0	1.0	1.0

Note for Table 1: Ascorbic acid, citric acid, and thiourea are far in excess (2,700–55,000 times larger than the iron concentration), whereas the ferene is in excess rendering ~1:220–320 ratio. The calculations were for practical examples, e.g., 50 $\mu L$ of iron standard (100 $\mu {g}$/dL) added to 200 $\mu L$ of total assay reagents in the optimized method, 36 $\mu L$ of the same iron standard was added to 214 $\mu L$ of total assay reagents in the original method. 30 $\mu L$ of 100 $\mu {g}$/dL iron sample was delivered to the dry sensing channel in the sensor strip method.

### C. Common Spectrophotometric Features and Test Sample Sources

All spectrophotometric measurements for the original and optimized reference methods were performed with 96-well plate in a Spectra Max M5 spectrophotometer at 590 nm, using $250~\mu \text{l}$ final test volumes, which rendered a path length of 0.6 cm.

Iron standards were made fresh from Fe(III) nitrate nonahydrate crystals in 0.5 M nitric acid solution using high-intensity shakers for 15 minutes to ensure iron crystals were completely dissolved. Calibration curves for spectrophotometric measurements (original and optimized refence methods) and new sensor strips measurements were obtained from analysis of iron standards: a blank, 25, 50, 100, 150, and $300~\mu \text{g}$/dL, covering the physiologically relevant total iron levels.

In addition, eight venous blood samples were obtained via consent (from each subject via Arizona State University’s IRB study protocol (STUDY00008255). All the samples were processed for serum and used for intra-laboratory validation. Two of the eight samples were sent to LabCorp for inter-laboratory validation. A total of 20 independent draws and tests were performed for intra-laboratory validation.

All experiments were conducted by the same technician, on the same instrumentation over the course of several months.

### D. Specificity of the Iron Detection Reaction

In order to study the selectivity of the iron detection reaction, we tested the response to several potential serum interferent analytes using the optimized reference method. The interferents’ analytes included glucose (140 mg/dl), creatinine (1.2 mg/dl), uric acid (7 mg/dl), potassium chloride (20 mg/dl), sodium chloride (333 mg/dl), and urea (20 mg/dl). The concentration of interferents was chosen to be the highest concentration values that could be found in a healthy human body blood [28]–[31].

### E. Kinetic Investigation of the Optimized Method

In order to develop a better knowledge of the iron detection reaction under the conditions of our optimized reference method, we studied the reaction kinetics, using 50 and $100~\mu \text{g}$/dL iron standards in combination with 2 and 4 mM ferene concentrations. We used time profile of absorbances and their corresponding numerical derivatives to determine the reaction order and rate constants. This analysis enabled the rational selection of iron detection reaction times, which is critical for the development of iron detection on dry sensor strips.

### F. Sensor Strip Design

**FIGURE 1.**
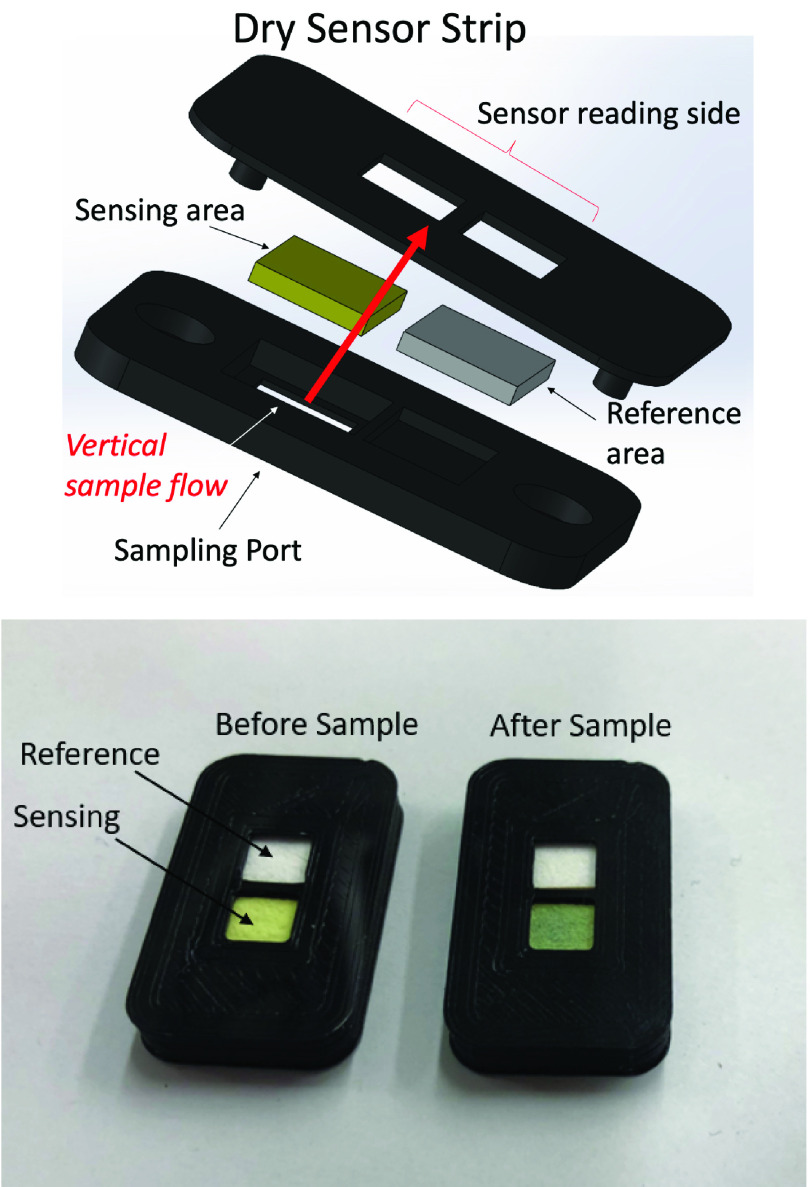
Top: 3D printed design and assembly of the sensor strip. Bottom: Sensor strip with sensing and reference areas before *(left)* and 5 min after *(right)* delivering a $30~\mu \text{L}$ sample.

We focused on iron analysis from serum with the goal of developing a novel assay for accurate, sensitive, and reproducible detection of iron. The assay consists of an accurate, storable, and robust dry sensor strip in a vertical flow design with the aim of iron detection time of five minutes to imitate current state of commercial medical devices. **Figure 1, top part** shows the 3D sensor design, indicating the sample delivery port and the sensing side, as well as assembly. The sensor strip has a sensing area and a reference area (**Figure 1, bottom part**). The sensing pad is made of a dry, porous, and absorbent nitrocellulose blotting filter paper impregnated with all reagents, which resulted in a built-in capacity to drive a $30~\mu \text{L}$ sample by capillary forces without spilling, and rapidly separating large components such as proteins from soluble ions [22]. Reagent A strips Fe(III) from transferrin, reduces Fe(III) to Fe(II), and chelates potential interferents such as Cu(II), whereas reagent B (ferene) chelates Fe(II) to form the colored complex. The reference pad comprises the same (white) material as the sensing pad, but has no reagents. In summary, the sensing pad facilitates processing of iron, containment for chemical reactions, and production of color change, whereas the reference pad does not accept sample and is present for reference lighting conditions during the measurement. To prevent sample leakage from sensing area to reference area, the two areas were separated with a 0.5mm-thick polylactic acid wall. The sample does no contact the reference area. There is only one sampling port that hosts the sampling membrane. The color change is read via reflection of light resulting from the white LED incident light on the sensor surface. The sensor housing was designed in collaboration with SolidWorks. The sensor was 3D printed using an Ultimaker 3 printer requiring Polylactic acid (PLA) feed polymer.

In order to estimate the final reagent concentration ratios in the dry sensor strip, 14 dried sensing channel blotting filter papers were weighed prior to and after reagent impregnation, calculating weight differences to determine the weight of reagents. Assuming the reagents in total (ascorbic acid, citric acid, thiourea, and ferene) were absorbed maintaining their solution mass concentration ratio, we estimated the reagents’ concentrations once rehydration occurred upon wetting with $30~\mu \text{L}$ samples. The results of the final local concentrations in the sensor are shown in **Table 1**. To obtain good fabrication reproducibility, the membranes should be: 1- dipped for 20 seconds, 2- dried at 45 degrees Celsius for 2 hours, and 3- cut with a laser cutter exactly with the same dimensions. This procedure resulted in a CV of 6.15% (N = 14).

### G. Sensor Strip Stability to Heat Exposure

In order to explore the stability of the sensor to heat exposure, we performed accelerated tests by placing the sensors in sealed aluminized Mylar^®^ bags (from Sorbent Systems) and storing them in an oven at 50°C versus control sensors at room temperature of 20°C for 2 days. Based on a predicted accelerated test algorithm, the accelerated aging factor (AAF) defined as the ratio of the room temperature estimated time and the accelerated aging time was as follows [32]:

\begin{equation*} \mathrm {AAF}=2^{\mathrm {X}}\tag{1}\end{equation*}

with

\begin{equation*} \mathrm {X}=\frac {\mathrm {T}_{\mathrm {heat}}-\mathrm {T}_{\mathrm {room}}}{10}\tag{2}\end{equation*}

representing 16 days of accelerated aging with no loss in sensitivity.

### H. Smartphone Mount

**FIGURE 2.**
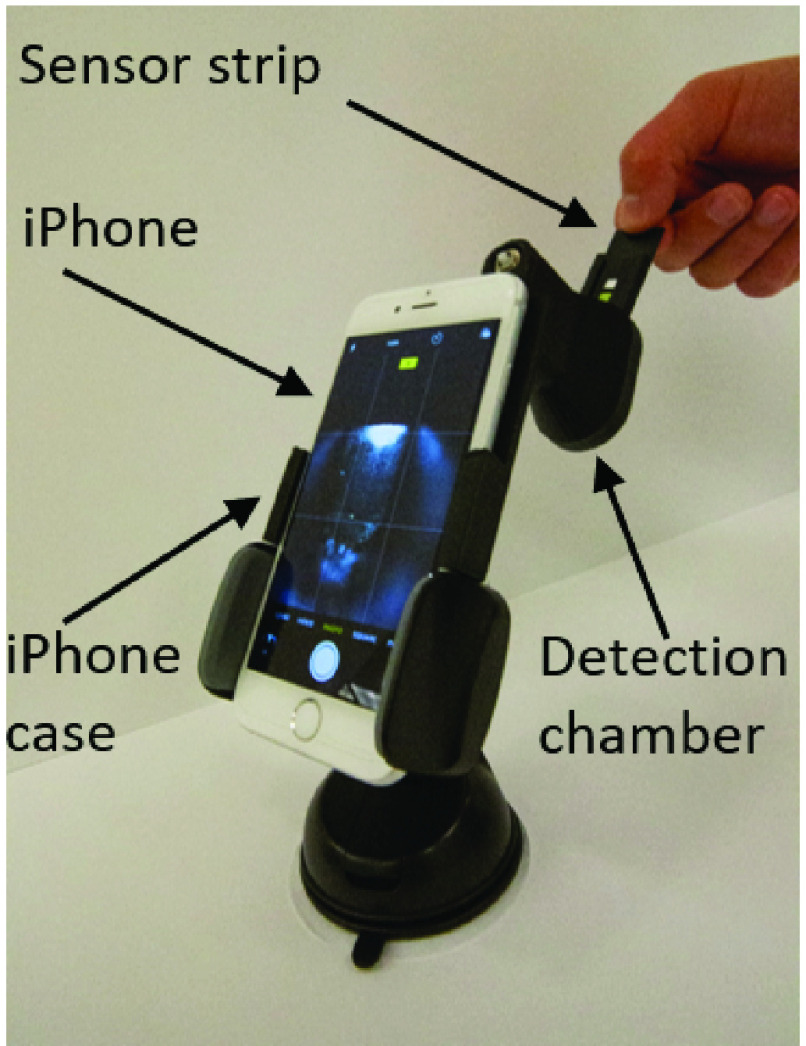
iPhone mounted appropriate distance from sensor strip. All material is 3D printed to control position and LED lighting, for very little cost. The image shows the user inserting the sensor strip into the chamber.

To secure the position of the sensor strip and to consistently control the smartphone camera’s exposure to lighting, a smartphone mount was designed with SolidWorks and printed with the Ultimaker 3. As shown in **Figure 2**, the iPhone is mounted at an appropriate focal distance from the sensor strip. A battery-powered LED light source circuit is mounted inside the chamber, requiring strategically placed lighting diffusers.

**FIGURE 3.**
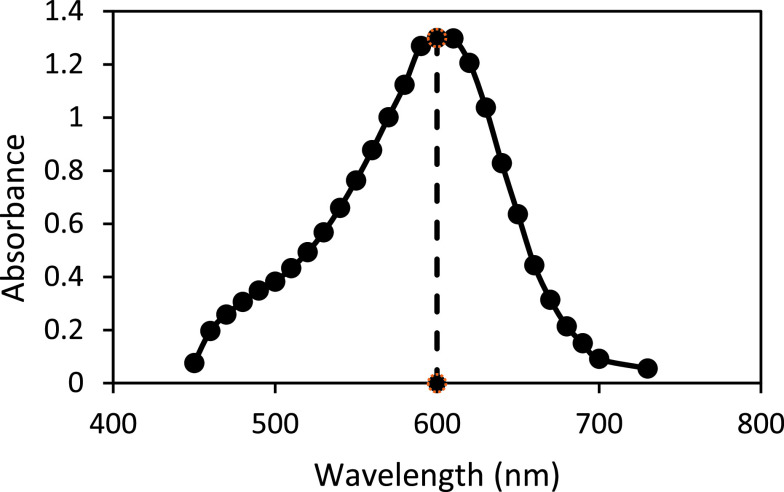
Spectral analysis of the iron complex indicating an excitation wavelengths ~ 600 nm.

### I. Smartphone Measurement of Iron on the Sensing Strip

Apple’s iPhone provides high quality imaging hardware and software useful in precision colorimetry, offering at least a 12-megapixel iSight camera at 1.5 micron pixels, autofocus, $f$/2.2 aperture, hybrid IR filter, exposure control, and in our case, with the flash turned OFF [33]. Color changes from the sensing and reference areas were electronically captured (images) and analyzed utilizing an in-house iOS app developed for the iPhone and device (mount, chamber, and strips). Deconvolution of the images’ Red, Green, and Blue (RGB) components was compared with that of a publicly available ImageJ software (requiring a personal computer with plug and play camera) to analyze how well the smartphone could capture high quality images, process colors quickly, and be easily programmed for a user-friendly experience. To this end, a wide variety of colors were printed on paper by an RGB generator and then systematically analyzed by both technologies. The resulting RGB intensities were then compared for agreement (see supporting information section). Next, iron standards ($25~\mu \text{g}$/dL to $300~\mu \text{g}$/dL) were used to generate a calibration curve, followed by human serum samples with sensing and reference areas read by both technologies and absorbance signals calculated using the following equation:

\begin{equation*} \mathbf {Absolute~absorbance}=-\log \left ({\frac {{\mathrm {\mathbf {I }}}_{\mathbf {sensing~channel}}}{{\mathrm {\mathbf {I }}}_{\mathbf {reference ~channel}}} }\right)\tag{3}\end{equation*}

where $I =$ intensity rendered from RGB component deconvolution of the colors.

### J. Environmental Conditions’ Effect on Sensitivity

We studied the sensitivity of the sensor strips at low and high temperature and relative humidity conditions ranging from 10°C to 51°C and 10% to 72%.

### K. Statistical Analysis

#### 1) Comparison Between Methods

We created calibration curves from known iron standards for both the “original reference” method, the “optimized reference” method and our novel assay. The slopes and linear correlation coefficients between the different methods were compared.

#### 2) Intra-Method Precision

The level of precision of each method was evaluated via coefficients of variation (CV %) reported as follows:

\begin{equation*} \mathbf {CV}\left ({{\%} }\right)=\frac {\mathrm {\mathbf {standard~deviation~of~slope}}}{\mathbf {average~of ~slope}}\tag{4}\end{equation*}

where “slope” represented the sensitivity (absorbance change vs. known iron concentration).

#### 3) Intra-Laboratory Validation Method

A correlation curve between the iron concentration values assessed in serum samples by the optimized reference method and our novel assay were compared and the linear relationship determined the average accuracy of the novel assay proposed here. In addition, we performed a Bland-Altman plot analysis for the purpose of showing additional characteristics of maximum error between the afore-mentioned methods.

#### 4) Inter-Laboratory Validation Method

the results of blood samples from LabCorp and our novel assay were compared in two of the blood samples, and the differences between the methods were determined as percentage errors.

## III. Results and Discussion

### A. Optimized Method for Iron Measurement

In order to confirm the reported maximum absorption wavelength of the iron complex, spectral analysis of the Fe(II)– ferene complex was performed. A single sharp peak with a maximum absorbance ~ 600 nm was observed, which was consistent with current literature and the chosen reference method (590 nm) [24], [25].

**FIGURE 4.**
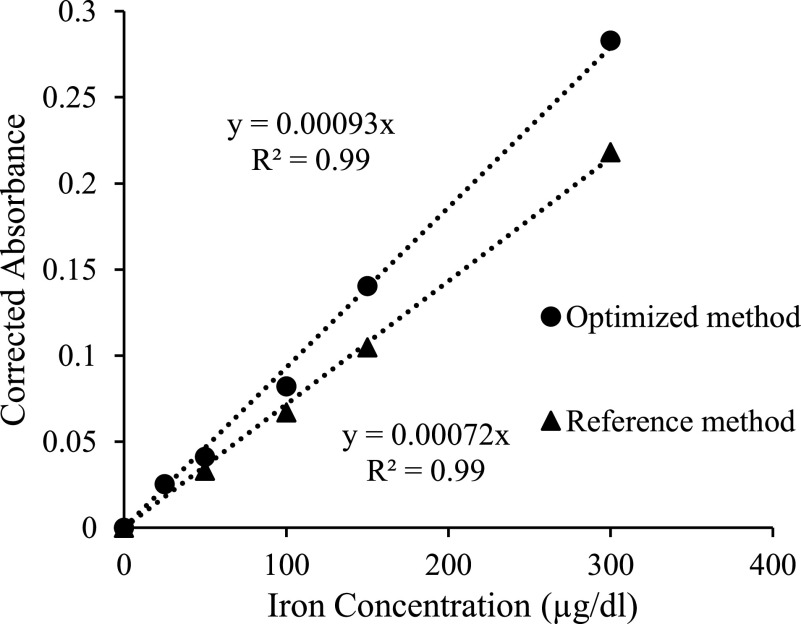
Calibration curves compared. The reference spectrophotometric method is a 5:1:1 volume ratio (reagents A to ferene to iron standards), giving a slope of 0.00072. Optimized method with 3:1:1 volume ratio, increased sensitivity by 30%, providing a slope of 0.00093.

Calibration curves based on the absorbance changes at 590 nm as a function of the iron standard concentrations were made, presenting a linear dependent signal as a function of the known iron concentration. The sensitivities of the optimized and original reference methods were 0.00093 and 0.00072 a.u./$\mu \text{g}$/dL) respectively, with a regression coefficient of 0.99 for both methods (**Figure 4**). The CVs of the optimized and original methods for the iron standards were 2.2%, and 3.7%, respectively. Thus, the sensitivity of the optimized method was 30% greater than the original method while the CV of the optimized method was 40% smaller than the original, giving a higher-quality method against which to test the results of smartphone analysis of total iron in our human subjects’ serum samples.

### B. Measuring the Effect of Protein Precipitation from Real Samples

**FIGURE 5.**
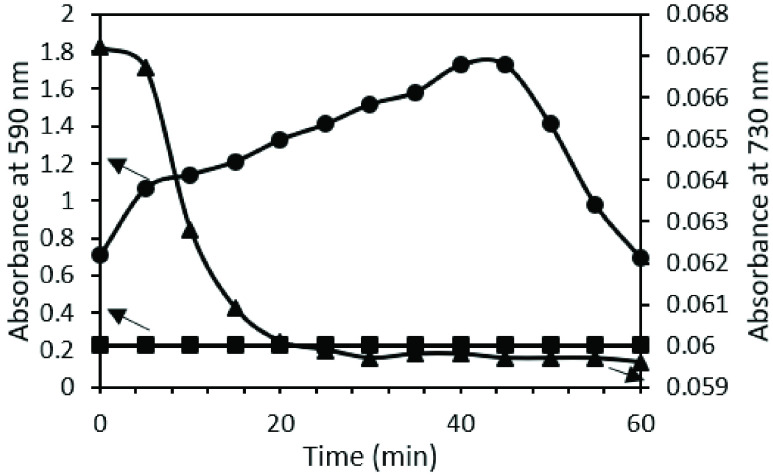
Left y-axis: Known serum sample with $231~\mu \text{g}$/dL total iron absorbance change at 590 nm over time using a) the original reference method (•) and b) the optimized method (squares). Right y-axis: Same known serum sample ($231~\mu \text{g}$/dL iron) at 730 nm (non-iron complex absorbing) over time reveals a “turbidimetry profile” that results from the original method (triangles $\blacktriangle $).

While the original reference method performs well on iron standards, it is highly variable with serum samples, resulting in unpredictable fluctuations throughout 60 minutes incubation. **Figure 5** shows an example profile of iron detection absorbance changes at 590 nm vs time for a known serum sample of $231~\mu \text{g}$/dL total iron, for both the original reference and optimized reference method. At minute 10, the original method (circles) is already four times greater (and growing) than that of the optimized method (squares). To investigate whether the turbidity in the original method’s solutions contributed to the unpredictable and greater absorbance values, we measured at a non-absorbing wavelength for the iron complex, 730 nm (triangles). These non-zero absorbance values and high fluctuations were indeed indications of interfering turbidity. As a result, we removed surfactant for all future use as a reference method, as well as for the sensing strips design. In addition to sensitivity improvement, removing the surfactant shortened the detection time from 30 minutes (as indicated in the protocol of the original reference method) to 2 minutes.

### C. Specificity of the Optimized Reference Method

**FIGURE 6.**
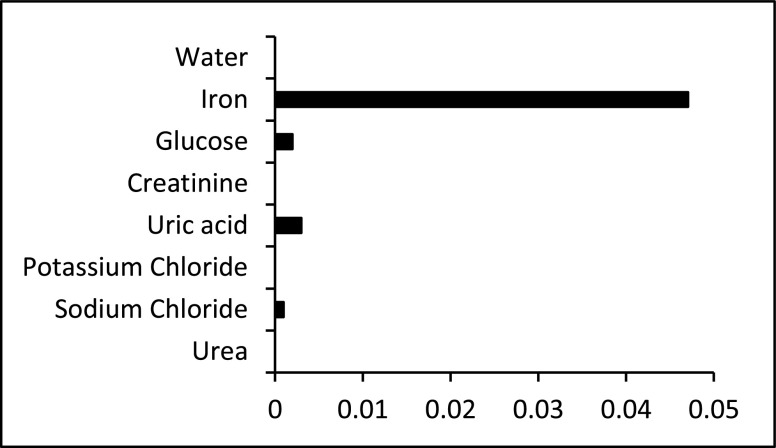
Specificity test. Comparison of the absorbance values to iron standard (0.05 mg/dL), water, glucose (140 mg/dl), creatinine (1.2 mg/dl), uric acid (7 mg/dl), potassium chloride (20 mg/dl), sodium chloride (333 mg/dl), and urea (20 mg/dl).

Since the sensor strips of the new assay were built based on the reagent’s concentration used in the optimized reference method, the same method was used to study the specificity of the iron detection reaction. The response to $50~\mu \text{g}$/dL of iron at 590 nm was compared to potential interferents, each concentration representing the high end of concentration for human blood. **Figure 6** shows that interfering signals from the most common and abundant molecules in blood, including glucose, creatinine, uric acid, potassium chloride, sodium chloride and urea. The responses from the interferents were negligible.

### D. Kinetics Results Using Optimized Method

In order to gather more insights on the iron detection reaction, we performed spectrophotometric kinetic studies. In addition, we determined the molar extinction coefficient of the Fe(II) - ferene complex (COM) for the optimized reference method conditions as an initial step. The resulting extinction coefficient was 33,366 L.cm$^{-1}$.mol$^{-1}$, utilizing the slope of the calibration curve for the optimized method, a path length of 0.6 cm, and the Beer-Lambert law. The assessed value was similar to that reported in the literature (34,500 L.cm$^{-1}$.mol) [15], [26]. The rate of the Fe(II) - complex (COM) formation in presence of ascorbic acid (AA), oxidizing to dehydroascorbic acid (DAA) ($k$) was analyzed as follows:

\begin{align*}&\hspace {-2pc} \text {2Fe}(\text {III}) + \text {H-AA-H} + \text {6ferene}.\xrightarrow {k} 2[\text {Fe(II)} - \text {3ferene}] \\&\qquad \qquad \qquad \qquad \qquad \quad \qquad \qquad {+ \text {AA}+ \text {H}_{2} }\end{align*}

Considering that the AA concentration was in excess, the overall reaction rate (k’) was simplified to:

\begin{equation*} \text {Fe}(\text {III}) + \text {3ferene}{\mathop {\rightarrow }^{k'}_{{{\it AA~{\textit{in}} ~{\textit{excess}}}}}} \text {Fe}(\text {II}) - \text {3ferene}\end{equation*}

Under the above-described conditions, the reaction order for iron ($\alpha$), and ferene ($\beta$) was determined according to the following rate law:

\begin{equation*} \frac {\mathrm {d}\left [{ \mathrm {COM} }\right]}{\mathrm {dt}}=\mathrm {k}^{\mathrm {'}}\left [{ \mathrm {F}\mathrm {e}^{3+} }\right]^{\alpha }\left [{ \mathrm {ferene} }\right]^{\beta }\end{equation*}

Details are shown in the Supplementary information and summarized in **Table 2**, which indicated almost a first order reaction for iron ion, and almost a second order reaction order for ferene.

**TABLE 2:** Kinetic Parameters of Reaction Rates (k, k’) and Reaction Order for Iron ($\alpha$), and Ferene ($\beta$) From Studies of the Ferrous Complex Formation in Presence of Ascorbic Acid (AA) in Excess

Parameter	Value
Alpha ($\alpha$)	1.2
Beta ($\beta$)	2.3
$\text{k}^{'}$ (mM$^{{-1.47}}\text{s}^{-1}$)	0.089
k (mM$^{{-2.47}}\,\,\text{s}^{-1}$)	0.0026

### E. Novel Assay With Dry Sensor Strip

**FIGURE 7.**
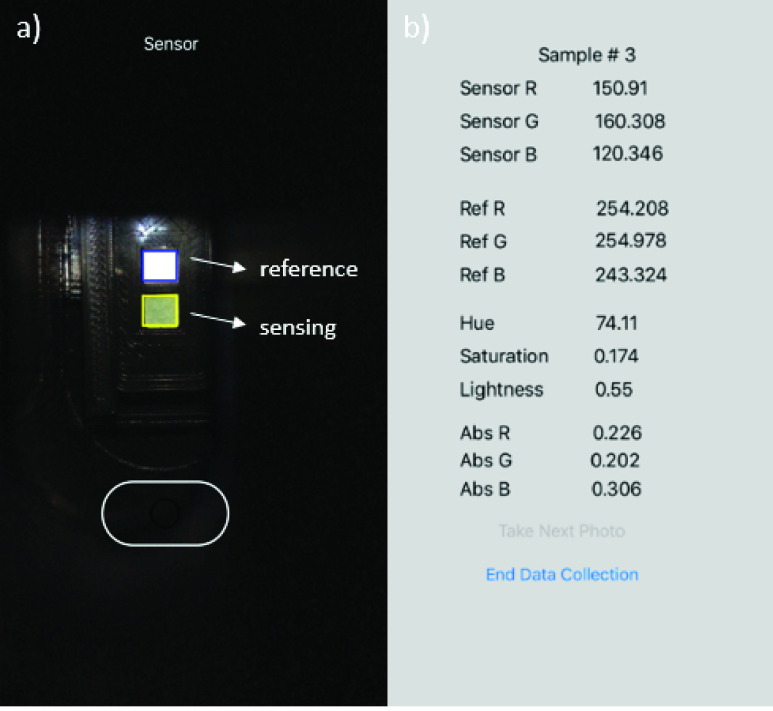
Smartphone application user interface showing a) camera’s view of the sensing and reference area on the dry sensor strip and b) iOS app output screenshot after sensor strip image is taken.

**FIGURE 8.**
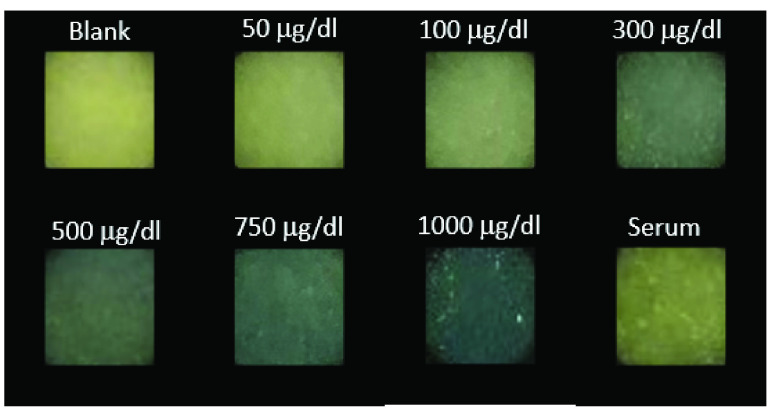
Smartphone application’s images of the sensor strip’s sensing area upon exposure to increasing iron ion standard concentrations and to serum.

First, we tested operational performance of the in-house built iPhone App using ImageJ software as a reference method. Linear relationships of 1.01, 1.06, and 0.90 with null y-axis intercepts (and regression coefficients of 0.98, 0.99, 0.97, respectively) for the Red, Green, and Blue component signal correlations between the two methods were obtained (see Supplementary Information section, *Smartphone app validation*). The results indicated that the App was accurately assessing the RGB component signals from the images. Next, iron standards were applied to the sensor strips and inserted into the smartphone reader, after which images were taken and analyzed by the smartphone app. In parallel, the same sensor strips were processed by the software ImageJ. **Figure 7** shows screenshots of the iPhone App for the sensor strip during the image capture (a), and result output following RGB analysis (b). **Figure 8** shows a close up feature of the sensing channel of different sensor strips exposed to increasing iron standard concentrations, and serum.

**FIGURE 9.**
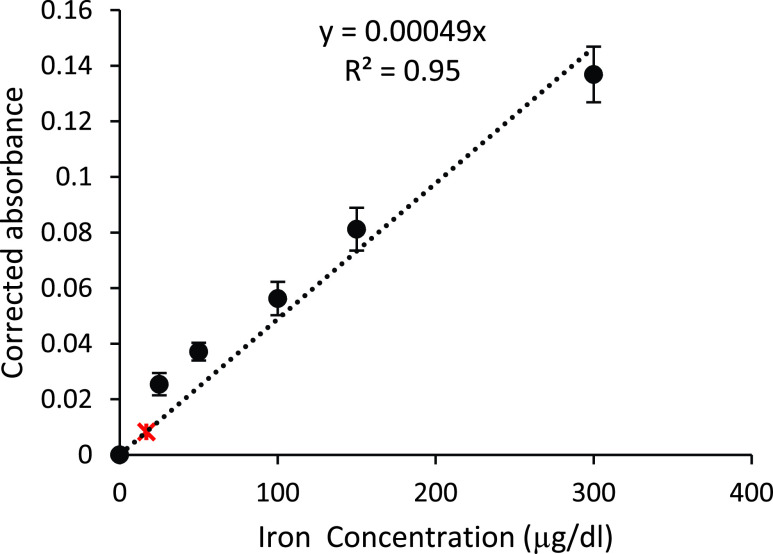
New assay calibration curve from 25–$300~\mu \text{g}$/dL resulted in a slope of 0.00049, comparable to the optimized reference method 0.00093 in Figure 4. Average values are marked with +/−1 standard deviation. The estimated limit of detection was $16.5~\mu \text{g}$/dL, marked with a red asterisk.

Thirty calibration curves of 25 to $300~\mu \text{g}$/dL were obtained using the new assay and compared to those calibration curves obtained with ImageJ processing. The comparison rendered a negligible difference. The Red component consistently produced the most sensitive data, with a slope of 0.00049, $\text{r}^{2} =0.96$, and CV of 10.5% (**Figure 9**), compared to Green (0.00032, $\text{r}^{2} =0.97$), whereas Blue was not sensitive to the iron concentrations. Red was thus chosen as the sole sensing signal for producing the calibration curve for the new assay. In addition, an estimated detection limit (LoD) of $16.5~\mu \text{g}$/dL total iron concentration was calculated from the assessed sensitivity, and by assuming a signal equal to 3 times the noise level from 30 blank samples (marked with a red asterisk at $16.5~\mu \text{g}$/dL in **Figure 9**) [34], [35].

#### 1) Intra-Laboratory Validation

**FIGURE 10.**
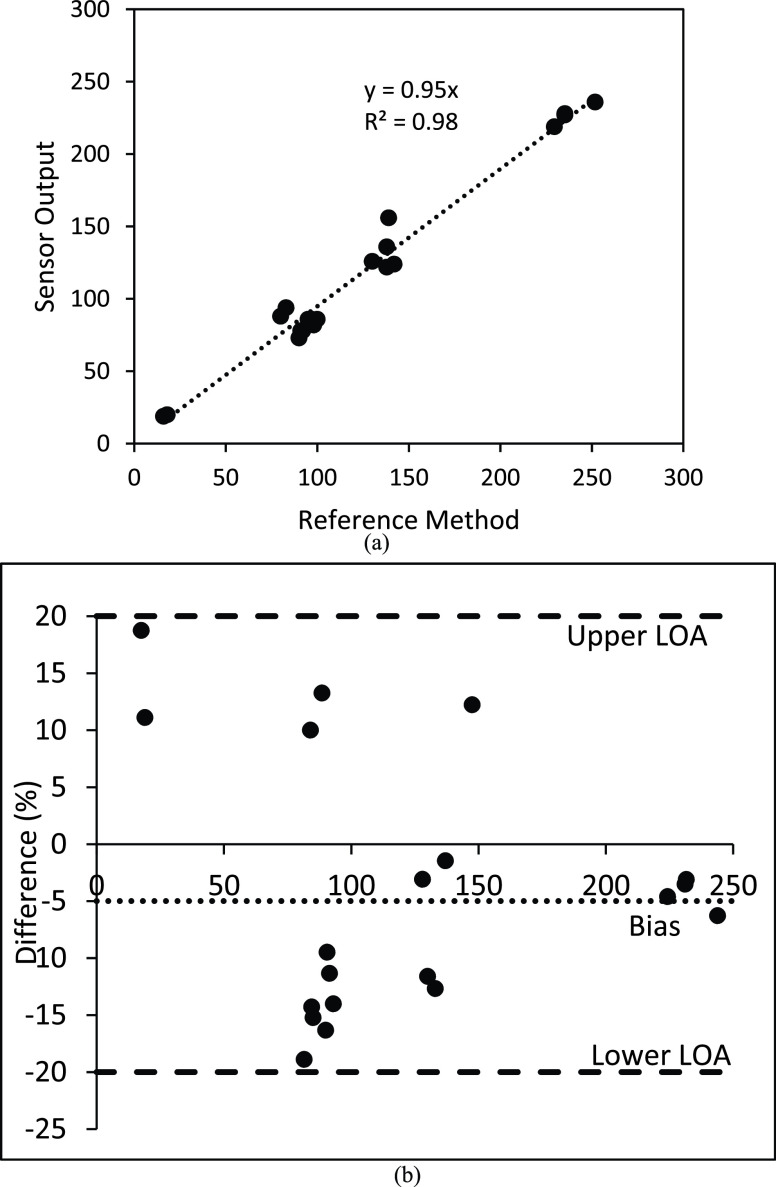
A. iPhone readings of dry sensor strips (x-axis) versus internal “optimized” reference method (n = 20). B. Percent Bland-Altman plot showed a bias of −4% and limits of agreements of 20% and −20% respectively (n = 20).

**Figure 10A** shows the correlation analysis between the output iron concentration values from serum samples between the novel assay and the modified reference method with a slope of 0.95 and regression coefficient of 0.98 for a total of 20 test. Further, **Figure 10B** shows the corresponding Bland-Altman plot that revealed a bias of −4% with lower and upper limits of agreements (95% CI) of 20%.

The Bland-Altman plot showed two important features. On one hand, a negative bias of 4% indicates that the device output is generally accurate and there is an overall accuracy with 4% error. Second, the highest mismatch between the experimental and reference methods were recorded for low iron concentrations with maximum percent differences of 20% while this difference creeps down to 5% at high iron concentrations. For screening purposes, these mismatches can be accepted. For example, in the extreme case where a person’s serum iron is 20 ug/dl, the device output will range between 16 ug/dl −24 ug/dl; and thus, will accurately predict that the person is at risk of iron deficiency.

#### 2) Inter-Laboratory Validation

For inter-laboratory validation, two of the venous blood samples were sent to LabCorp and reported to be $231~\mu \text{g}$/dL and $203~\mu \text{g}$/dL respectively. The same samples were analysed with the optimized method and we arrive at a result of $238~\pm ~18~\mu \text{g}$/dL and $206~\mu \text{g}$/dL $\pm 10\mu \text{g}$/dL, which confirmed our decision to eliminate the surfactant.

### F. Environmental Operational Conditions of Use

**FIGURE 11.**
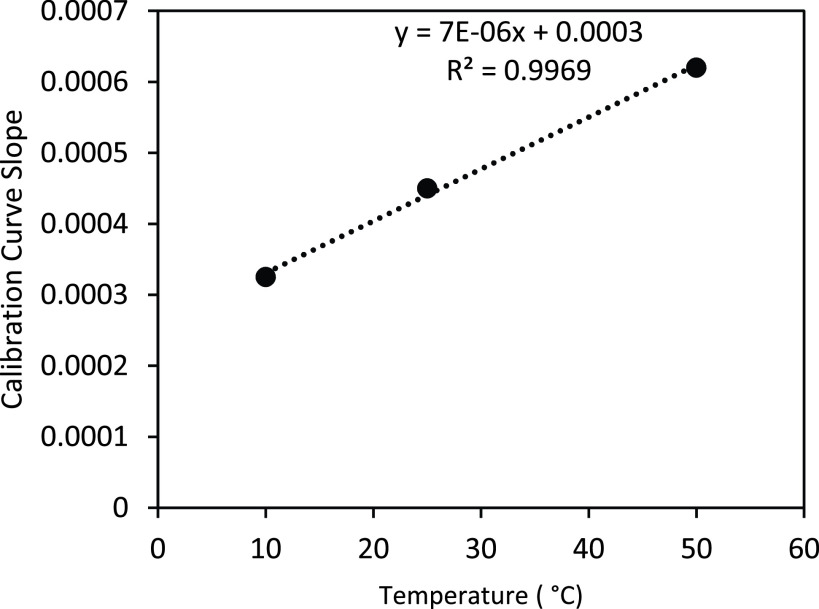
Calibration curve sensitivity plot with temperature for the following ranges (10°C – 50°C).

**FIGURE 12.**
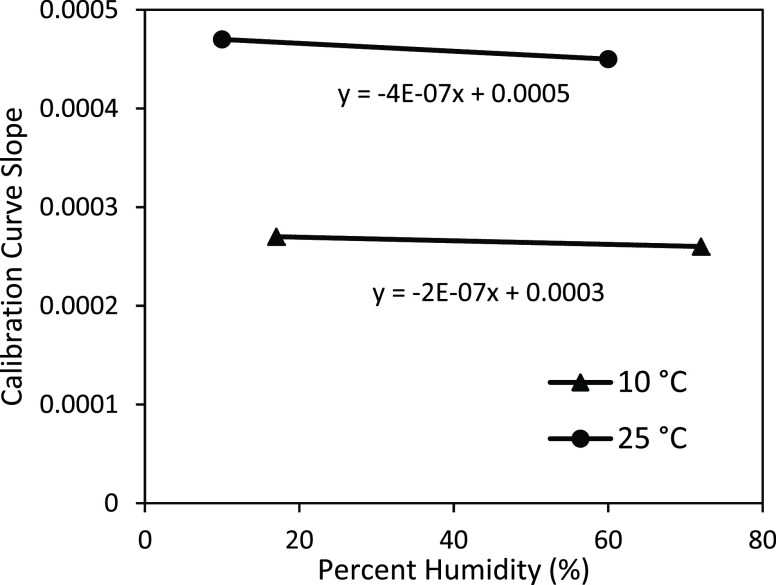
Calibration curve sensitivity plot with percent humidity (17% and 72%) at 10°C and (10% and 60%) at 25°C.

All results shown above were assessed at 23–25 °C. The study of the effect of environmental operational conditions on the sensitivity of novel assay allowed us to conclude that the temperature affects sensitivity at $7\times 10^{-6}$ a.u / ($\mu \text{g}$/dL)/°C (**Figure 11**), while the environmental relative humidity does not influence significantly the sensitivity. **Figure 12** shows slopes of $4\times 10^{-7}$ a.u / ($\mu \text{g}$/dL) / % humidity at 10 °C and $- 2\times 10^{-7}$ a.u / ($\mu \text{g}$/dL) / % humidity at 25 °C). This indicates that the novel assay practiced in parallel with environment temperature assessment could be applied to assess iron concentration at any temperature with the calibration temperature range between 10°C – 50°C (tested environmental operational conditions).

### G. Cost Analysis of the Novel Assay

The cost to implement the novel assay is summarized in **Table 3**. Assuming the smartphone is already owned by the tester, the assay cost includes two components: 1) a one-time investment to acquire the smartphone mount, and 2) the sensor strip. The fabrication of the smartphone mount is ~$13 (considering retail components price and associated time pro-rated labor). The sensor strip cost is <$0.06. Although the current version of the assay takes the centrifugation step for serum separation from blood into account, in the future, the sensor strip will accommodate whole blood from human capillary puncture, which will not add substantial cost to the sensor strip. Therefore, we seek to drive down the cost of CLIA lab-based total iron measurements at the current rate of $25.00 to under $0.06 per test.

**TABLE 3:** Cost Breakdown of New Assay

Smartphone mount	Cost ($)
LED circuit & diffusers	8
Power cord to charge device	3
3D printed chamber	2
Total investment cost	$13
Each Sensor Strip	
Plastic	0.04
Sensor pads	0.0024
Reagents	0.017
Total investment cost	<0.06

## IV. Conclusion

In summary, we present the development of a new assay, which includes a dry sensor, a smartphone mount, and an app. This assay has similar detection accuracy as our in-house modified reference spectrophotometric method, and a third-party laboratory (LabCorp). Given the strong need for inexpensive, less invasive, and rapid screening and monitoring of iron levels in humans at risk for iron deficiency or overload, the new assay is a significant contribution to the solution.

Inspired by successful glucose control for management of diseases (Type I and Type II diabetes) and by former experiences of implementing technologies connected to mobile devices [36], [37], we have completed several steps toward creating a new point-of-care total iron measurement assay for prevention or intervention in human iron deficiency or toxicity. First, we have created a reference method that can be used to validate iron detection; second, we have tested the iron chemistry for serum processing in an inexpensive dry sensor strip; third, an in-house smartphone mount and an App has enabled rapid reading of the sensor strip chemistry for testing accuracy and reproducibility; and last, our methodical approach to optimizing chemistry and color change processing has led to knowledge of the detection reaction kinetics. Our experimental data confirmed the new assay offered a linear relationship between serum iron and absorbance in physiologically normal ranges.

The novel assay with the dry sensor strip and smartphone mount, and App was sensitive to iron detection with a dynamic range of 25– $300~\mu \text{g}$/dL, sensitivity of 0.00049 a.u/$\mu \text{g}$/dL, coefficient of variation (CV) of 10.5%, and estimated detection limit of $\sim 15~\mu \text{g}$/dL. These analytical specifications are useful for predicting iron deficiency and overloads. The optimized reference method had a sensitivity of 0.00093 a.u/$\mu \text{g}$/dL and CV of 2.2%. The correlation of serum iron concentrations in the intra-laboratory testing between the optimized reference method and the novel assay rendered a slope of 0.95, and a regression coefficient of 0.98, suggesting that the new assay is accurate. Last, we performed spectrophotometric validation of the iron detection reaction kinetics for the test conditions to reveal almost a first order reaction for iron ion, and almost a second order reaction order for ferene.

In summary, the new assay provided satisfactory accuracy results in our intra- and inter- laboratory validations and provided promising features of mobility and low-cost manufacturing for global healthcare settings. Future work is oriented to full integration of the on-sensor strip with whole blood processing capabilities, and total iron binding capacity tests.

## Conflicts of Interest

There are no conflicts to declare.
